# Surgical stabilization for symptomatic carpometacarpal hypermobility; a randomized comparison of a dorsal and a volar technique and a cohort of the volar technique

**DOI:** 10.1007/s00238-016-1212-8

**Published:** 2016-07-13

**Authors:** Kim Robin Spekreijse, Guus Maarten Vermeulen, Thybout M. Moojen, Harm P. Slijper, Steven E. R. Hovius, Ruud W. Selles, Reinier Feitz

**Affiliations:** 1 Division of Plastic, Reconstructive and Hand surgery, Erasmus Medical Center, Rotterdam, the Netherlands; 2 Division of Hand surgery, Xpert Clinic, Hilversum, the Netherlands

**Keywords:** Carpometacarpal, Thumb, Stabilization, Hypermobility

## Abstract

**Background:**

Hypermobility of the first carpometacarpal joint is mostly surgically treated with a volar approached stabilization by Eaton, but recent studies indicate the importance of the dorsoradial and intermetacarpal ligaments (DRL and IML) for carpometacarpal joint stability. The aim of this study was to compare a dorsal and volar technique for primary carpometacarpal hypermobility regarding pain and functional outcome.

**Methods:**

Patients with non-degenerative, painful carpometacarpal hypermobility were included and were randomly assigned to either the volar technique using the FCR, or a dorsal technique using the ECRL. After premature termination of the trial, we followed all patients treated with the volar approach. Pain, strength, and ADL function using DASH and Michigan Hand Questionnaires (MHQ) were measured at baseline and 3 and 12 months after surgery.

**Results:**

After including 16 patients, the randomized trial comparing the volar and dorsal technique was terminated because of significant increased pain in the dorsal group. Although none of the other outcome measures were significant in the underpowered comparison, in line with the pain scores, all variables showed a trend towards a worse outcome in the dorsal group. Between 2009 and 2012, 57 thumbs were surgically stabilized. We found significant better pain and MHQ scores, and after 1 year improved grip and key pinch strength. Patients returned to work within 8 (±7) weeks, of which 85 % in their original job.

**Conclusions:**

Surgical stabilization of the thumb is an effective method for patients suffering from hypermobility regarding pain, daily function, and strength. We recommend a volar approach.

Level of Evidence: Level I, therapeutic study

## Introduction

Joint laxity or hypermobility of the first carpometacarpal (CMC) joint is a common disorder. In some cases, this generalized joint hypermobility combined with subluxation of the joint can cause severe pain and can result in osteoarthritis of the first CMC joint later in life [1–4]. This symptomatic thumb base hypermobility is also seen in systemic diseases, like Ehlers Danlos and Down syndrome. Furthermore, it is common in young women with generalized joint hypermobility or postmenopausal women with an early stage of osteoarthritis (Eaton & Glickell stage I) [5, 6]. This study focuses on the surgical treatment of young, female patients, suffering from generalized joint laxity without an underlying specific (systematic) disease. These patients commonly present with pain, functional problems, instability, and loss of strength. Loss of strength is most evident during pinch, grasping movements, and unscrewing actions.

Since it is often assumed that the deep oblique anterior ligament (dOAL), also called the beak ligament, provides the most joint stability, the most commonly used surgical procedures in the treatment of symptomatic hypermobility of the first CMC joint are based on supporting or reinforcing the function of this volar beak ligament [1, 7]. The technique described by Eaton and Littler [8], using a volar approach, is the most commonly used technique for reinforcing the function of this volar beak ligament. The technique supports the beak ligament and the intermetacarpal ligament (IML) with the flexor carpi radialis (FCR) tendon in this volar technique and is reported to be effective in this specific patient group for pain reduction and for prevention of CMC degeneration [8, 9].

However, more recent anatomical studies indicate that the dorsoradial ligament (DRL) and the IML are more important for CMC joint stability than the beak ligament [10]. Based on this, it may be better to reconstruct the unstable first CMC joint by reinforcing the function of these two ligaments. Since the IML and DRL might be better approachable from the dorsal side of the joint, a dorsal approach was described in 1945 by Eggers [11], using the extensor carpi radialis longus (ECRL) tendon to support the DRL and IML.

To our knowledge, a volar approach, focused on reinforcing mainly the function of the volar beak ligament, has never been compared with a dorsal approach, focused on reinforcing the function of the DRL and IML. Therefore, the aim of this study was to directly compare a dorsal and volar approach in a randomized controlled trial for the surgical treatment of symptomatic CMC hypermobility in patients with primary hypermobility in terms of pain, complications, and functional outcome.

## Methods, pt. 1

### Study design

The study was conducted in 2008 and 2009 as a randomized single-blinded comparative trial of a volar versus dorsal stabilization for CMC joint laxity in the Department of Hand and Wrist Surgery, Diakonessenhuis, Zeist, the Netherlands, with approval of the local scientific committee. High pain scores in the dorsal group lead to prematurely termination of the randomized controlled trial (RCT) (see Results); hence, a cohort study of all patients treated with the volar technique was initiated.

After informed consent was obtained, patients were included if they had (1) severe pain during daily use of their thumb, (2) first metacarpal subluxation during physical examination, and (3) a failed conservative treatment such as splinting and physical therapy. X-rays were taken and examined by an independent radiologist to exclude early stages of osteoarthritis (Eaton & Glickel stages I and II). We excluded patients with previous invasive treatments (e.g., injections or surgery).

### Surgical techniques

**Fig. 1:**
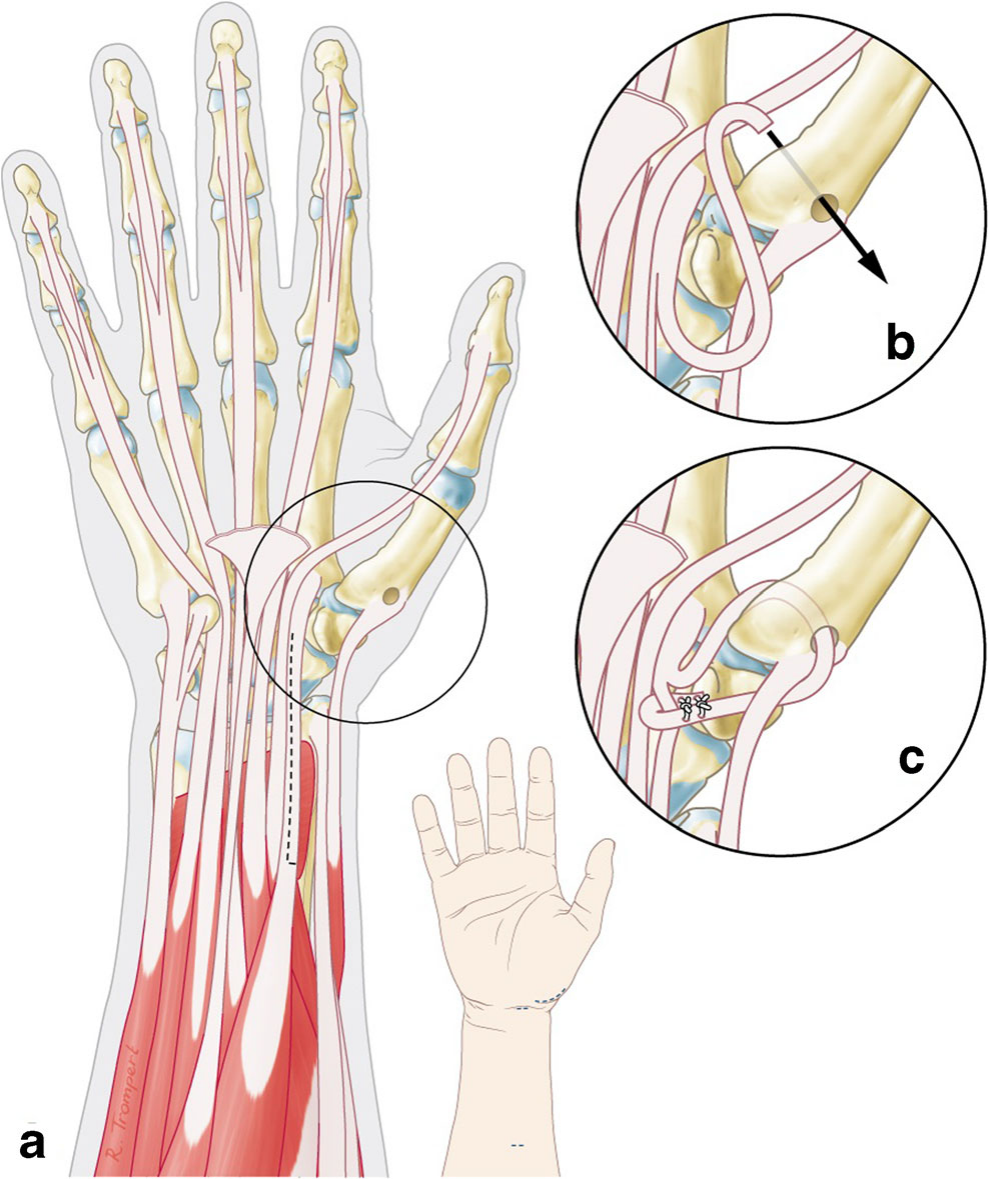
CMC stabilization with a volar approach **a** using a slip of the FCR tendon, **b** passed through a bone tunnel in the first metacarpal and **c** tied down to its insertion

For the volar technique, we slightly modified the technique of Eaton and Littler [8], which uses a slip of the flexor carpi radialis (FCR) to reconstruct the beak ligament using a Wagner incision. Originally, the tendon slip is taken back to insertion of the remaining FCR and attached to itself to provide volar-radial stability. In the modified technique, the FCR tendon was slip in the traditional way with a small incision proximal, and distally just proximal of the distal pole of scaphoid. The tendon is passed under the thenar muscles to the radial side of the CMC joint. A formal Wagner approach is not needed; just a small incision over the radial side of the CMC joint is used to get access to the base of the first metacarpal bone. By splitting the tendon more proximal than was described by Eaton and Littler, the direction of the force is more oblique. After this, a hole is drilled in the first metacarpal and the tendon is passed from volar to dorsal and sutured to the abductor pollicis longus and then passed around itself and tied down (see Fig. 1). No K-wires were used for immobilization. We used a short period of cast splinting of 2 weeks after which a protective splinting was provided for 8 weeks and active range of motion exercises were started under supervision of specialized hand therapists.

**Fig. 2:**
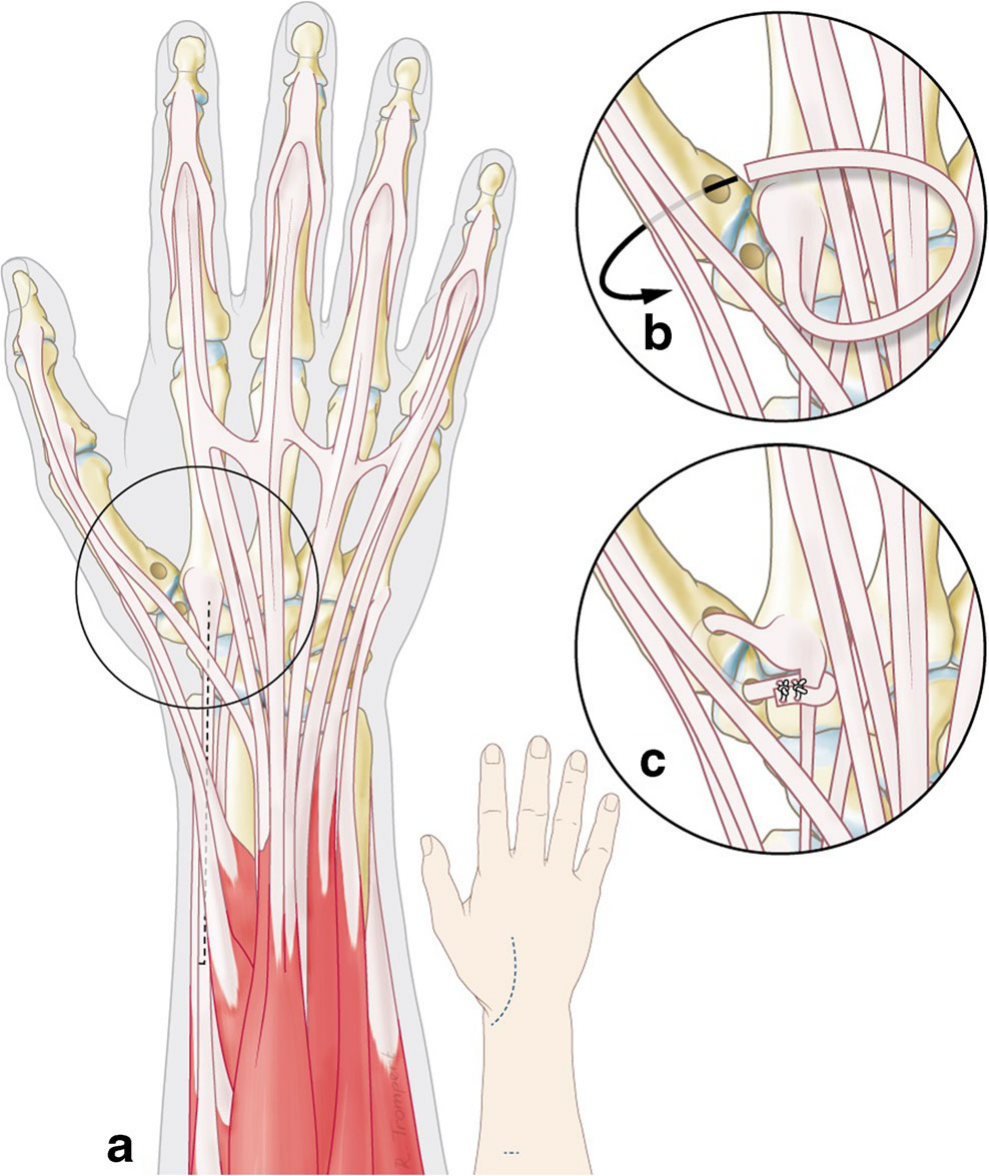
CMC stabilization with a dorsal approach **a** using a slip of the ECRL tendon, **b** passed through a bone tunnel in the first metacarpal and back through a bone tunnel in the trapezium and **c** tied down to its insertion

For the dorsal technique, an incision of approximately 5 cm was made from the insertion of the ECRL at the base of second metacarpal towards the radial styloid, preserving the radial superficial nerve. After opening the capsule and creating a bone tunnel in the base of the first metacarpal, a slip of the ECRL was passed through this tunnel, back through a tunnel in the trapezium bone and reattached to itself at the base of the second metacarpal (see Fig. 2). Both the DRL and the IML were thereby reconstructed and stability was restored. As in the volar approach, K-wires were not inserted to immobilize the joint. The postoperative protocol contained the same standard cast immobilization and hand therapy.

### Randomization

After consultation and inclusion in the trial, patients were randomly assigned to a surgeon; both surgeons had their preferable method so randomization was performed at this level. We chose an equal randomization type with the usage of opaque envelopes which were drawn by the doctor’s assistant after consultation, deciding which surgeon would be operating on this patient and therefore which type of technique would be used in the stabilization.

### Outcomes measures

Medical history and demographic data of each patient were recorded at first presentation. Independent hand therapists performed measurements at baseline and 3 and 12 months after surgery.

Our primary outcome measurements were pain and hand function. Pain was measured using a visual analogue scale (VAS). In the RCT, function was measured with the “Disabilities of the Arm, Shoulder and Hand” (DASH) questionnaire. The DASH score is a frequently used questionnaire for the function of the upper limb (0 = no disability, 100 = completely disabled in arm, hand, and shoulder) [12].

As secondary outcome measures, grip strength was measured using a hydraulic hand dynamometer and tip, key, and tripod pinch were measured using a pinch gauge meter. In addition, we evaluated overall patient satisfaction with the treatment using a five-level Likert scale and patients were asked 1 year after surgery if they would choose for the same treatment again under the same circumstances. Complications were recorded for a period of 12 months for each patient.

### Statistical analysis

To compare the two treatment groups, a generalized estimating equations (GEE) approach was used. Using both group allocation and time interval (3 months and 12 months versus preoperative) as predictors, the comparison between the dorsal and volar technique was compared in three dimensions; between both groups, over time and the group × time interaction effect, which evaluates if the change over time is different between both groups. Evaluation of treatment satisfaction and complications were tested using independent *t* testing, Mann–Whitney *U* tests, and chi-square statistics, and a *P* value smaller than 0.05 was considered statistically significant.

## Results, pt. 1

Between 2008 and 2009, 16 patients were enrolled in the randomized trial. Table 1 shows the baseline characteristics of both groups. After these 16 patients, however, we found that patients treated with the dorsal approach had a slow and more painful recovery (when compared to the volar approach) reporting back to the treating physician in the outpatient clinic. Therefore, we performed an interim analysis which indicated a significant difference in change of pain scores at 3 months (see Table 2), with a decreased average pain score in the volar group and an increased pain in the dorsal group. Based on this, the trial was prematurely terminated. Although none of the other outcome measures were significant in the underpowered comparison, in line with the pain scores, all these variables showed a trend towards a worse outcome in the dorsal approach. For complications, see Table 3. In the dorsal group, two patients suffered from tendinitis (ECRL and morbus de Quervain), recovering after steroid injection. In the volar group, one patient had postoperative scar tenderness and one patient had an extensive infection, requiring three revision surgeries combined with split skin grafting.

**Table 1:** Baseline characteristics of both the randomized controlled trial and the cohort

	Randomized controlled trial		Cohort
	Dorsal approach	Volar approach	Volar approach
No. of patients	8	8	57
Sex (no. female)	7	8	51
Age (years)	35.9 (SD 5)	35.9 (SD 13)	34.0 (SD 13)
Dominance (no. right handed)	7	8	53
Operated hand (no. right)	5	6	35
Dominant hand operated (*n*)	6	6	35
Smoking (*n*)	1	0	5

**Table 2:** Estimated means and standard errors of outcome measures and their change over time compared in both groups of the randomized controlled trial

	Treatment group	Mean (SD SE)			Difference in change over 3 months (mean SD SE)	*P* value	Difference in change over 12 months (mean SD SE)	*P* value
		0 months	3 months	12 months	Short-term		Long-term	
DASH (0–100)	Dorsal	34.8 (SD 11.1)	29.7 (SD 9.2)	45.9 (SD 7.8)	−5.4 (SD 15.9)	0.734	−24.8 (SD 16.1)	0.124
	Volar	38.0 (SD 6.0)	27.5 (SD 5.5)	24.3 (SD 7.0)				
VAS pain (0–100)	Dorsal	30.3 (SD 2.9)	43.0 (SD 7.9)	–	−45.7 (SD 15.4)	0.003	–	–
	Volar	58.5 (SD 11.0)	25.5 (SD 5.5)	–				
Grip (kg)	Dorsal	17.0 (SD 1.6)	21.0 (SD 1.6)	22.1 (SD 3.0)	1.0 (SD 5.9)	0.862	5.9 (SD 4.6)	0.199
	Volar	18.3 (SD 3.4)	23.4 (SD 2.5)	29.4 (SD 1.8)				
Tip pinch (kg)	Dorsal	2.4 (SD 0.3)	2.7 (SD 0.3)	3.3 (SD 0.3)	0.6 (SD 0.6)	0.311	0.2 (SD 0.7)	0.747
	Volar	2.7 (SD 0.5)	3.6 (SD 0.3)	3.8 (SD 0.3)				
Tripod pinch (kg)	Dorsal	3.2 (SD 0.4)	4.5 (SD 1.0)	4.4 (SD 0.4)	−0.7 (SD 1.5)	0.656	0.2 (SD 1.1)	0.888
	Volar	3.6 (SD 0.9)	4.3 (SD 0.4)	4.9 (SD 0.4)				
Key pinch (kg)	Dorsal	4.6 (SD 0.4)	4.9 (SD 0.4)	5.2 (SD 0.6)	1.7 (SD 1.0)	0.084	1.4 (SD 1.0)	0.177
	Volar	4.0 (SD 0.7)	6.0 (SD 0.3)	6.0 (SD 0.4)				
Satisfaction (1–5 Likert scale^a^)	Dorsal	–	–	4.75 (SD 0.4)	–			0.806
	Volar	–	–	4.5 (SD 1.0)				
Same operation again? (Y/N)	Dorsal	–	–	88 %	–			1.000
	Volar	–	–	88 %				

Satisfaction and “same operation again” were asked only 12 months after surgery so no difference over time was measured

*SE* standard error, *DASH* Disabilities of the Arm, Shoulder and Hand, *VAS* visual analogue scale

^a^1–5 Likert scale very poor; poor; acceptable; good; very good

**Table 3:** Complications recorded in both groups of the RCT and in the cohort

	Randomized controlled trial		Cohort
	Dorsal approach	Volar approach	Volar approach
Mild (no treatment)			
Scar tenderness		1	4
Sensory disturbances			5
Moderate-severe (treatment)			
Tendinitis (treated with steroids)	2		1
Mild CRPS type I			
Neuroma			
Infection		1	1

## Conclusions, pt. 1

Because of a significant increase in pain after surgical stabilization for CMC joint laxity, using a dorsal approach, compared to an improvement in both pain and function using a volar approach, we abandoned the dorsal approach and continued only using the volar approach.

## Methods, pt. 2

After termination of the RCT, all patients with symptomatic primary hypermobility were treated with the volar technique (by the surgical technique as described earlier) and were prospectively followed as a cohort, based on the same inclusion criteria as described for the randomized trial part.

### Outcome measures

The same primary and secondary outcome measures were used as in the randomized trial. In addition, we used the “Michigan Hand Questionnaire” (MHQ; 0 = poorest function, 100 = ideal function) to allow evaluating both hands separately [13].

### Statistical analysis

For the retrospective analysis of the cohort, we also used a GEE analysis to evaluate the treatment effect over time. Because not all patients already were at 12 months after surgery, a missing value analysis was performed using the Little’s MCAR (chi-square) test, a *P* value higher than 0.05 means that the characteristics in the groups with complete data are not significantly different from the group that did not achieved full completion of all measurements.

## Results, pt. 2

Between 2008 and 2014, 54 patients (57 thumbs) were treated. Patients were predominantly women between 20 and 40 years old (Table 1). The *P* value of the Little’s test was 1.000 indicating that missing values were missing completely at random. Therefore, all cases were used in the analysis.

Overall, we found that the volar approach significantly reduced pain and improved the MHQ score at 3 and 12 months after surgery (see Table 4). Despite the pain reduction, on average, a residual pain remained at 12 months (mean 24 on a score from 0 to 100). All strength measures were also significantly improved at 12 months, while outcomes at 3 months were not significantly different from baseline. Active range of motion was not significantly changed at 12 months, except for a small but significant improvement in Kapandji score. At 3 months, a decreased metacarpophalangeal (MCP) range of motion was found, which may be related to joint stiffness after a period of immobilization.

**Table 4:** Estimated means and standard errors of outcome measures and their change over time in the cohort

	Mean (SD SE)			Difference in change over 3 months (mean—95%CI)	*P* value	Difference in change over 12 months (mean—95%CI)	*P* value
	0 months	3 months	12 months	Short-term		Long-term	
MHQ (0–100)	54.4 (SD 2.1)	63.6 (SD 1.8)	70.8 (SD 4.2)	9.3 (4.2–14.3)	<0.001	16.4 (7.5–25.3)	<0.001
VAS pain (0–100)	61.3 (SD 3.1)	32.9 (SD 3.2)	23.5 (SD 5.9)	−28.4 (−37.4–−19.4)	<0.001	−37.8 (−60.0–−24.7)	<0.001
Grip (kg)	22.0 (SD 1.4)	22.5 (SD 1.5)	29.0 (SD .4)	0.5 (−2.7–3.8)	0.755	7.0 (3.6–10.3)	<0.001
Tip pinch (kg)	2.8 (SD 0.2)	3.1 (SD 0.2)	3.8 (SD 0.3)	0.3 (−0.2–0.8)	0.205	1.0 (0.4–1.6)	0.001
Tripod pinch (kg)	3.8 (SD 0.3)	4.0 (SD 0.3)	5.1 (SD 0.4)	0.1 (−0.5–0.8)	0.675	1.3 (0.4–2.1)	0.003
Key pinch (kg)	4.5 (SD 0.3)	4.7 (SD 0.3)	6.5 (SD 0.4)	0.1 (−0.6–0.8)	0.715	1.9 (1.1–2.7)	<0.001
Flexion IP-joint (°)	70.8 (SD 2.8)	69.2 (SD 2.3)	70.2 (SD 6.1)	−1.5 (−7.9–4.9)	0.640	−0.5 (−13.1–12.0)	0.934
Extension IP-joint (°)	−16.9 (SD 2.7)	−13.9 (SD 3.5)	−21.4 (SD 7.0)	3.0 (−5.7–11.7)	0.497	−4.4 (−19.3–10.4)	0.557
Flexion MCP-joint (°)	57.1 (SD 2.8)	51.0 (SD 2.2)	52.9 (SD 7.3)	−6.1 (−11.9–−0.3)	0.038	−4.1 (−18.9–10.6)	0.582
Extension MCP-joint (°)	−4.4 (SD 2.7)	26.7 (SD 5.7)	−8.1 (SD 3.6)	31.1 (19.2–43.1)	<0.001	−3.8 (−11.8–4.2)	0.357
CMC Opposition (Kapandji) (0–10)	9.0 (SD 0.2)	8.6 (SD 0.2)	9.6 (SD 0.2)	−0.4 (−1.0–0.1)	0.113	0.6 (0.1–1.0)	0.012
CMC Abduction (IMD) (mm)	7.4 (SD 12.4)	52.8 (SD 4.2)	55.1 (SD 4.5)	−20.6 (−46.2–5.0)	0.115	−18.3 (−44.0–7.3)	0.161
Satisfaction (1–5 Likert scale^a^)	–	–	4.2 (SD 0.9)				
Same operation again? (Y/N)	–	–	90.9 %				
Return to work (weeks)	–	–	7.8 (SD 6.9)				

Satisfaction, “same operation again” and return to work were asked only 12 months after surgery so no difference over time was measured

*SE* standard error, *CI* confidence interval, *MHQ* Michigan Hand Questionnaire, *VAS* visual analogue scale

^a^1–5 Likert scale very poor; poor; acceptable; good; very good

The patients could return to work or activities within an average of eight (SD 7) weeks of which 85 % with the same intensity as preoperative. Twelve months after surgery, 6 % rated their treatment as “poor,” 13 % as “average,” 34 % as “good,” and 47 % as “excellent.” Ninety percent of the patients reported that they would choose this surgery again. The complication rate was 19 % of which two patients had major complications; one patient had revision surgery after 3 years because of development of osteoarthritis, another patient as earlier mentioned had multiple revision surgeries because of an extensive infection (Table 3). Of the minor complications, four patients reported scar tenderness for which silicone therapy was started; five patients had transient sensory disturbances like tingling and allodynia.

## Discussion

Due to the premature termination of the randomized controlled trial, statistical power of this study was low. Despite of this, the interim analysis indicating a significant difference in change in pain scores at 3 months between both groups, with a decreased average pain score in the volar group and an increased pain in the dorsal group. Due to the small number of patients included in the RCT, no other conclusions can be drawn because of the absence of other statistical differences between groups. Another limitation of this study is the relatively high percentage of missing data in the cohort study. An important reason for this missing data was that data were measured by the patients’ own hand therapist, some of whom were not integrated in our hand clinic. Furthermore, we missed 12 months pain scores in the RCT part of the study. As a result, we are not able to compare how pain scores would develop in both groups over time. An important limitation of the cohort study was the lack of a control group.

To our knowledge, a direct comparison of a dorsal and volar approach for surgical stabilization of the thumb CMC joint has not been reported and so far; no data have been reported in literature on the surgical outcome of a purely dorsal technique. Eggers [11] described the dorsal approach with a slip of the ECRL in 1945 for the first time but did not present patient results. Biddulph [13] hypothesized that harvesting of the FCR would be more invasive and extensive than with a dorsal, more superficial approach. However, during this procedure, the CMC joint is still opened by a volar approach in contrast to the dorsal harvesting of the tendon. Twenty-five patients with a follow-up between 5 and 10 years were treated with this procedure, showing restored pinch strength and excellent results were reported but without showing actual results. Brunelli et al. [14] described a technique in 1989 with the accessory APL tendon passed through drill holes of the first and second metacarpal bones and attached to the ECRL insertion. They showed good to very good results in 12 patients. However, this technique with a dorsal approach is only focusing on restoring the IML without any support of the DRL.

In contrast to surgical stabilization for symptomatic CMC hypermobility, for trapeziectomy in osteoarthritis, results of dorsally approached ligament reconstruction can be found as well as a comparison with a volar approach. Ritchie et al. [15] found similar results when they compared trapeziectomy with a dorsal and volar approach in the treatment of basal thumb osteoarthritis. They concluded that the volar approach resulted in better key pinch, joint mobility, and patient’s satisfaction, while the dorsal approach group had more scar-related complications and therefore they also preferred a volar approach. Illarramendi et al. [16] reported 84 % pain relief and 89 % satisfaction in patients with osteoarthritis and dorsally approach surgical stabilizations with the ECRL after trapeziectomy, which seems to be in contrast with our findings [16].

Despite the volar technique [8] is over 40 years old and although many modifications have been made, it is still widely used and the success of the volar approach in our study is in line with other studies on the volar approach. In 2001, Lane et al. performed surgery on 37 cases of non-arthritic CMC instability, using Eaton and Littler’s [8] original technique with an average follow-up of 5.2 years; 67 % rated “excellent” on the Likert scale and 30 % rated “good,” and all patients improved in pinch strength [2]. Van Giffen et al. [17] performed Eaton’s volar technique on 18 thumbs (of which six thumbs were post-traumatic) with overall good results and at a mean follow-up of 5 reported a mean DASH of 23.2 and VAS of 3.1, very similar to our results. Freedman et al.[4] reported on 19 patients (24 thumbs, of which 26 % post-traumatic) with Eaton original technique with good or excellent result in 71 % after a mean follow-up of 7 years but pain in 71 % of the cases. Because it was unclear how these pain scores were measured, we cannot compare these results directly to ours.

Our finding that a dorsal surgical approach results in less optimal outcome than a volar approach may be related to a richer innervation of the dorsal part of the joint. The superficial branch of the radial nerve plays a major role in the sensibility of the CMC joint [18], embedded in the dorsal capsule. We postulate that cutting the capsule to enter the joint can cause pain and develop scar tissue. Recent literature suggests that the DRL is also the most important stabilizer of the CMC joint [1, 19] and Mobargha et al. [20] recently showed that the DRL has a richer innervation than the volar dOAL, indicating this ligament is very important for proprioception and stabilization. Based on this, it could be that a dorsal approach creates more instability and more damage to joint proprioception and leads to more pain due to damage of the DRL.

In summary, the prematurely terminated trial shows a significant increase in pain in the dorsal technique treated group whereas the volar technique group had pain reduction at follow-up. In addition, the large cohort study of the volar technique indicates that patients with symptomatic CMC hypermobility can be treated with a stabilization of this joint, using a volar approach, leading to pain relief and good functional outcome. Based on our results, therefore, we do not recommend a dorsal approach in this specific patient group.
